# Supplementary material for: Discovery and application of insertion-deletion (INDEL) polymorphisms for QTL mapping of early life-history traits in Atlantic salmon
Source: BMC Genomics. 2010 Mar 8;11:156. doi: 10.1186/1471-2164-11-156 (PMC2838853; doi:10.1186/1471-2164-11-156)
Supplement: Additional file 2 — Information on developed 76 locus single-run INDEL panel in Atlantic salmon. Information on fluorescence labeling, primer concentrations, PCR pooling and links to alignments, INDEL motifs and GENESCAN (Burge and Karlin 1997) predictions of genes/exons are available in html format. [file 1471-2164-11-156-S2.ZIP › Additionalfile2/snpsummary7583.html]

```
Cluster 2252 Contig 2

prev  Summary    Contig List  next
```

Size of Consensus sequence = 712

Number of sequences = 11

Minimum redundancy = 4

Key

A gi|117832993|gb|EG905689.1|EG905689 EST\_ssal\_evf\_8277 ssalevf mixed\_tissue Salmo salar cDNA Salmo salar cDNA clone ssal\_evf\_509\_160\_fwd 3', mRNA sequence  
B gi|24356294|gb|CA050124.1|CA050124 ssalsrkc002089 kidney Salmo salar cDNA, mRNA sequence  
C gi|25998377|gb|CA769122.1|CA769122 ssalsrkc005040 kidney Salmo salar cDNA, mRNA sequence  
D gi|29310867|gb|CB499641.1|CB499641 ssalsrkc014076 kidney Salmo salar cDNA, mRNA sequence  
E gi|25998367|gb|CA769112.1|CA769112 ssalsrkc007010 kidney Salmo salar cDNA, mRNA sequence  
F gi|25998349|gb|CA769094.1|CA769094 ssalsrkc003093 kidney Salmo salar cDNA, mRNA sequence  
G gi|29310913|gb|CB499687.1|CB499687 ssalsrkc018054 kidney Salmo salar cDNA, mRNA sequence  
H gi|29310925|gb|CB499699.1|CB499699 ssalsrkc018069 kidney Salmo salar cDNA, mRNA sequence  
I gi|117832994|gb|EG905690.1|EG905690 EST\_ssal\_evf\_8278 ssalevf mixed\_tissue Salmo salar cDNA Salmo salar cDNA clone ssal\_evf\_509\_160\_rev 5', mRNA sequence  
J gi|117851270|gb|EG923966.1|EG923966 EST\_ssal\_evf\_26725 ssalevf mixed\_tissue Salmo salar cDNA Salmo salar cDNA clone ssal\_evf\_535\_158\_rev 5', mRNA sequence  
K gi|117430369|gb|EG762593.1|EG762593 EST\_ssal\_sjb\_655 ssalsjb mixed\_tissue Salmo salar cDNA Salmo salar cDNA clone ssal\_sjb\_005\_043\_rev 5', mRNA sequence

2 SNPs detected

A B C D E F G H I J K  cosegregation weighted

574 - A A A A A A A - - -   2/2 100.00
575 - T T T T T T T - - -   2/2 100.00
